# Exploring Alternative Care Platforms for Symptomatic People in the Fight against the Ebola Virus Disease Outbreak

**DOI:** 10.29245/2578-3009/2023/S3.1106

**Published:** 2023-05-12

**Authors:** Nkechi G. Onyeneho, Ngozi Idemili Aronu, Ijeoma Igwe, Joseph Okeibunor, Tieman Diarra, Amadou Baïlo DIALLO, Bairo Hamadou, Barry Rodrigue, Mamoudou Harouna Djingarey, Zabulon Yoti, Michel N’da Dick, Konan Yao, Ibrahima Socé FALL, Abdou Salam Gueye

**Affiliations:** 1 University of Nigeria, Nsukka; 2 World Health Organization, Switzerland; 3 Independent Consultant, Mali; 4 Independent Public Health Expert, Niger

**Keywords:** Treatment center, Ebola treatment center, Temporary containment area, Home-based care, People with disease symptoms, Community accountability

## Abstract

Treatment centers (TCs) are the only locations designed to care for people with Ebola virus disease (EVD) symptoms. These people and their families are held at a TC as soon as they arrive at an Ebola treatment center (ETC); however, some people escape from TCs. This paper explored alternative care platforms for symptomatic people in the fight against the EVD outbreak in the Democratic Republic of Congo. Eight hundred randomly selected adults aged 18 years and above were surveyed with a uniform set of structured questionnaires. In-depth interviews were conducted with 20 community/opinion leaders, while focus group discussions were held with community members who were not involved in the questionnaire study. Our findings demonstrated that people who were suspected of having EVD preferred to be treated discreetly and at home, and were more willing to be tested at home than at a TC. People were afraid of being stigmatized if the TC exposed their admittance to the general public. This article proposes an alternative to the TCs. We suggest a temporary containment facility within the community, such as a room in the suspected person’s home. However, this requires negotiation between the response team and community members, with the latter having a significant responsibility in caring for their symptomatic relatives. The place or room for domestic temporary isolation should be chosen discreetly and placed far from the view of others. Community members will, thus, bear more responsibility for what happens while the patient is in isolation. The temporary containment area will assist in decentralizing the treatment of those with EVD symptoms. Its implementation will contribute to greater accountability of community members in the fight against EVD.

## Introduction

It is not always straightforward to offer support to people suffering from Ebola virus disease (EVD) symptoms in a treatment center (TC). Even when the facility is in the heart of the community, local community members say it is too visible^1^. Community members predominantly fear TCs because of stigmatization. Furthermore, there are rumors that TCs are a direct pathway to Ebola treatment centers (ETC). Community members believe that TCs are contaminated and expose sick people to the public. The lack of confidentiality at TCs is the leading cause of desertion. Some people flee the center after being admitted, while others leave even before being tested and diagnosed^2,3,4^.

The unwillingness of community members, as well as of those who are symptomatic, is rooted in the fear of stigmatization by other communities should they have a positive EVD diagnosis^5^. The visibility of TCs’ physical structure is also a problem; people believe this increases their exposure to others who have tested positive for EVD. Furthermore, TCs are perceived as a site that do not maintain confidentiality. Most community members do not perceive TCs’ role of relieving the burden on ETCs. Symptomatic people have expressed a need to be anonymous. Although they were not hostile to treatment, some of them requested that testing be carried out at their homes in a discreet manner. Rumors concerning the disease, the epidemic, and the response inform people’s fear of being admitted to a TC. This fear is not only experienced by those who are symptomatic, but also by their relatives. Once they are admitted to a TC, check-ups begin. TCs are perceived as a dehumanizing place that prioritizes examination and testing those who are potentially ill, obtaining results, and deciding on the future of their treatments—either to admit them and provide care or discharge them.

Throughout this research, conversations revealed community members’ anxiety related to TCs. Those who had been suspected to be ill reported that they would have preferred being examined at home. This article offers an alternative model of care to support people with EVD symptoms: a temporary containment area (TCA). A TCA would not only be one physical structure, but a decentralized care facility. Instead, a TCA would entail home-based care in a designated place with the agreement of the patient’s relatives.

This space should be determined in collaboration with family members, who would be responsible for the care of their relatives. Once the place is chosen within the patient’s home, it would be submitted to the health personnel for approval. Arrangements will be made to allow testing under the required conditions. The patient’s home will then become a TCA. This will reduce fear and stigma among community members, and this accountability will also prepare them for further care.

There are several advantages to this confinement place, such as discretion and lack of stigma.

A TCA will also provide decentralization of the support offered to people with symptoms. It requires the accountability and involvement of the family, just as in the case of home-based treatment of malaria. Our study aimed to explore alternative solutions for EVD testing and diagnosis. First, this study focused on the patient’s home as a potential place for temporary confinement. Then, we addressed the responsibility of community members in the use of their home as a place for temporary confinement.

## Study Design and Methods

### Study Design

This study was designed to explore and document experiences and lessons around the response to the 10^th^ EVD outbreak in the North Kivu and Ituri provinces of the Democratic Republic of Congo (DRC). A cross-sectional design with mixed methods and techniques of data collection was employed, which allowed multiple windows of data harvesting, as well as reaping the benefits of both quantitative and qualitative analysis. The use of this method guaranteed the integrity, robust interpretation, and conclusion that our evaluation warranted.

**Selection of Study Area and Population**: The study was carried out in the North Kivu and Ituri provinces where the 10^th^ EVD outbreak occurred in the DRC.

**Ituri** is one of the 26 provinces of the DRC. Its capital is the city of Bunia. The Ituri Rainforest is found in this area. It is located northeast of the Ituri River and on the western side of Lake Albert. Ituri is a region of high plateau (2000–5000 meters) that has a large tropical forest, but also the landscape of savannah. The district has rare fauna, including okapi, the national animal of the Congo. As for flora, an important species is Mangongo, whose leaves are used by the Mbuti to build their homes. The population is composed primarily of Alur, Hema, Lendu, Ngiti, Bira, and Ndo-Okebo, with differing figures on which one of the groups constitutes the largest percentage of the population in the province. The Mbuti, a pygmy ethnic group, reside primarily in the Ituri forest near the Okapi Wildlife Reserve, although some Mbuti have been forced into urban areas by deforestation, over-hunting, and violence. The Kilo-Moto gold mines are partly located in Ituri. In the beginning of the 21st century, petroleum reserves were found by Heritage Oil and Tullow Oil on the shores of Lake Albert.

**North Kivu** (French: *Nord-Kivu*) is a province bordering Lake Kivu in the eastern part of the DRC. Its capital is Goma. North Kivu borders the provinces of Ituri to the north, Tshopo to the northwest, Maniema to the southwest, and South Kivu to the south. To the east, it borders the countries of Uganda and Rwanda. The province consists of three cities—Goma, Butembo, and Beni—and six territories—Beni, Lubero, Masisi, Rutshuru, Nyiragongo, and Walikale. The province is home to the Virunga National Park, a World Heritage Site containing the endangered mountain gorillas. Except for the heightened insecurity and isolation due to rebel activities, North Kivu shares similar demographics with Ituri. The province is politically unstable and has been one of the flashpoints of the military conflicts in the region since 1998.

The **2018 or 10**^**th**^ **Kivu Ebola outbreak** began on August 1, 2018, when four confirmed cases tested positive for the Ebola virus in the eastern region of Kivu in the DRC^6,7^. The Kivu outbreak encompassed the Ituri Province, after the first case was confirmed on August 13^8^. This outbreak started just days after the end of the EVD in the DRC^9^.

The affected province and general area are currently undergoing a military conflict, which is hindering treatment and prevention efforts. The World Health Organization’s (WHO) Deputy Director-General for Emergency Preparedness and Response has described the combination of military conflict and civilian distress as a potential “perfect storm” that could lead to a rapid worsening of the outbreak^10^. Due to the deteriorating situation in North Kivu and surrounding areas, the WHO, on September 27, raised the risk assessment at the national and regional level from “high” to “very high”^10^.

The study population comprised adults aged ≥18 years living in the community, as well as the response team members. As of 2010, the estimated population of North Kivu was 5,767,945. An estimate of the population at 70% yielded 4,614,356 individuals. In North Kivu, considering its annual growth rate of 3.2%, the population in 2019 was estimated to be 7,658,406 and 5,360,884 for the general public and those aged ≥18 years, respectively. By contrast, as of 2005, the estimated population of Ituri was 4,037,561; an estimate of those aged ≥18 years at 70% yielded 2,968,865 individuals. In 2019, the population was estimated to be 6,275,305 and 4,392,714 for the public and those aged ≥18 years, respectively.

The response team consisted of over 10,000 persons who belonged to different response pillars, namely surveillance, risk communication, social anthropology, and vaccination. Others included infection prevention and control, treatment and care, safe and dignified burial, security, logistics, and administration, among others.

### Methods

The next phase of the study was conducted using qualitative methodology through in-depth interviews. This type of study design requires a strong focus on individual actors rather than state actors^11^.

### Techniques of Data Collection

A set of questions covering different thematic areas were developed to guide the discussions. The questions covered healthcare services in the community, awareness of, and practices regarding EVD, as well as an assessment of the different pillars of response interventions.

In-depth interviews (IDIs) were conducted in each community, where a focus group discussion (FGD) was carried out. The IDI was held with community/opinion leaders in the selected communities for response pillars. Interviews were used to explore people’s opinions, views, and attitudes regarding practices and insights into the outbreak and responses, including other socio-cultural factors that may influence individuals’ attitude toward the response. An FGD guide was used for the IDI, focusing on thematic areas of interest for the evaluation.

All interviews and discussions were tape-recorded, and detailed notes were taken simultaneously, including verbal citations. Tape-recorded interviews were transcribed according to standard rules. Observations were also recorded and, together with the discussion and interviews, triangulation was performed using the quantitative data to arrive at conclusions.

#### Training and Pilot Trials

All instruments were ***translated*** into Swahili and French, the common languages spoken by the communities, and back translated to English for clarity of meaning. In each province, ***10 research assistants*** with substantial experience in community interactive research and the use of qualitative and quantitative techniques, as well as cultural sensibility, were recruited and trained for three days in Beni. During another three days in Bunia, the study objectives and use of the instrument for data collection were explained to them. Training also included data entry into the ATLAS.ti template (qualitative data) and EPI INFO (quantitative data). The instruments were reviewed after training for clarity, understanding, and sensitivity. Each province had a ***supervisor*** who worked with the Principal Investigator on data quality monitoring, safety advisory, and ethical conduct of the research, including the management of informed consent procedures. The study was conducted first in Ituri, then in North Kivu. The lessons learned from Ituri were used to manage the process in North Kivu, which is comparatively more security- and logistics-challenged. The ***data analyst*** developed and pre-tested the template for data entry and analysis using the pilot test output. Given the short period of the study, data were collected using pencil and paper instead of an android device. Fieldwork took 20 days to complete in each province before the analysis and report writing were performed.

### Data Management

IDIs were transcribed from the audio recordings to text. All textual data were analyzed, using the ATLAS.ti software package, according to themes corresponding to the indicators in the quantitative data. Thereafter, data were triangulated during presentation to enable complementary and analogous interpretation.

Given the continuous analytical process involved in qualitative analysis, it is important to note that the initial analysis of the key informant interviews and FGDs informed the final development of the structured questionnaire to be used in the study. This further enhanced triangulation between the two sets of data to be collected. While the quantitative results gave us statistical conclusions, the qualitative results placed emphasis on what was actually said and provided illustrative quotes that added context and depth to the quantitative results.

### Ethical Considerations

The principle of do-no-harm was adhered to in this study. Informed study approval was obtained from the province, local administration, community, and households, while informed consent was obtained from all individuals that were involved in the study. The WHO/AFRO Ethics Review Committee gave ethical approval for this study. All researchers attended a mandatory training, which included substantial discussion of the ethical issues in research. Fifty percent of the research assistants were females, ensuring same sex interviews and moderation of the FGD sessions. The assistants were also trained and mandated to comply with child protection and gender sensitivity in the process of data collection and visits.

## Results

### Home as a Place for Temporary Containment

A civil servant in the Bunia health zone wanted to stay at home to be tested and remain there until his test results were confirmed. This case exemplifies the search for confidentiality in treatment. Checking-in at these places is considered by the broader community as a confirmation of a status that many community members would like to hide from others. While the solution proposed by the official was not part of the management strategies, especially for those with symptoms, it was desired by patients who needed to be tested.

One can then envision a TCA, which would not exist in a specific place well-known to all the community members and would be considered as the antechamber of an ETC. TCs help relieve the burden on ETCs. If everyone who arrived at an ETC had to be tested, it would rapidly exceed its capacity. A TC guarantees the isolation of the patient, avoiding contact with family members or anyone else who may interact with them in family, work, or social settings.

TCs have always been an important part of the support offered to patients. The aim of this study was not to discuss the importance of TCs and the guarantees they offer, but rather to explore alternative platforms for the management of people with EVD symptoms. The answer to this question is more prospective. When the question comes up in an interview, the answer is thrown back to the person asking it.

The answers are then to be found in facts and social events. Management is an activity developed and performed by healthcare professionals. All that is required from the relatives of symptomatic people is to help healthcare professionals do their job. Management has not always been easy given the unwillingness of relatives or community members. This aversion is rooted not only in perceptions and representations of the disease, but also in the lack of information for family or community members. Their accountability starts with information.

### Accountability of Community Members

The accountability of family and community members can ensure that each household becomes a place for temporary containment, creating an alternative place for diagnosis. However, this will require collaboration among the health workers responsible for diagnostics and care, and the patient’s family members. Decentralizing the TC into a space located in all communities will allow the healthcare personnel to test and diagnose suspected cases.

## Discussion

The name “treatment center” indicates that it is a place of treatment, and therefore a passage to another place. The fact that people who do not test positive will not move on to an ETC does not make the TC a potential place to exclude from the system. People fear entering the established care circuit, which causes them to hide, refuse isolation, or flee from the center^12^.

TCs are a costly structure. Replacing TCs with a more open and less obvious space, in family structures, will lead to a reduction in the expenses of construction and management. However, this will require families and communities to act as central pillars of medicine and of the response. This new structure will, therefore, contribute to the treatment of Ebola via community participation. Community accountability was evident, for example, in the treatment of onchocerciasis through the Community Directed Ivermectin Treatment (CDIT) strategy. CDIT was effective in controlling onchocerciasis^13^.

TCA will therefore be an alternative platform managed through partnership among the healthcare staff, families, and community members. However, this model of care must be tested and validated. TCAs can be developed through tests or implementation, as was the case for the Ivermectin treatment under community guidelines^14^.

Some literate people who presented with symptoms asked to stay with their families while being tested and waiting for diagnosis. The last of these requests were made based on the following:

- The desire to go unnoticed, which is motivated by the fear of TCs and especially by the need for more privacy.
- Fear of stigma: Staying among relatives is perceived as a way of escaping the gaze of others. This gaze is felt in many places before arriving at the TC and during the time spent there.
- The quest for safety: If TCs are a place for diagnosis, they can also be a place of exposure to the disease, and people can be infected by others while awaiting diagnosis.
- Discretion: The traffic for transportation to the TC and for response activities is not always appreciated by community members. Moreover, it is a way of revealing the events and does not ensure discretion.
- Moreover, there was evidence to support the feasibility of TCAs.
- The focus is not on the location, as the TCA will not have a determined location; the focus will be on strategy.
- This strategy requires the involvement of families and community members. They shall be informed and qualified to learn the conditions for implementing temporary confinement to protect all other family and community members. In the TCA, physical isolation may happen within the family.
- The division of responsibilities between family members and healthcare personnel: This is a home-based care approach. All the relatives or a specific member can be responsible. Thus, the person with symptoms will be assisted by a family member or a peer.
- The involvement of the families and communities offers a great opportunity for people to participate in treatment as they will be on familiar ground.
- The family and community will be at the heart of the new strategy. Their responsibility is not technical, but rather social. The family and community become accountable as the temporary shelter will require not only the provision of space but also a place for preventing the spread of EVD to other members.
- Negotiation will take place with the family and community for the implementation of temporary confinement.
- Temporary confinement will happen under the responsibility of the family and community. It is a family- and community-based model of care. The family manages the health of its members and even has a role in therapeutic choices. Therefore, this is not a new responsibility.
- Micro-caging, as practiced in Guinea, has proven to be an effective means of tracking contacts. Resorting to families for temporary containment offers the same opportunity.
- The use of temporary confinement also implies a relaxation in the screening and management system.
- Temporary containment will contribute to the community-based treatment of EVD. However, it will not be done under community guidelines as it requires medical expertise, especially regarding testing and diagnosing.
- Temporary containment will help engage the families and communities on matters related to the epidemic.
- Temporary confinement will assist in providing therapeutic support to the symptomatic person in a more accepted and discreet way.
- Community providers can be involved, if necessary, especially to reassure the person and provide moral and psychological support.

Home-based support is provided for malaria, and it has helped ease the burden on the healthcare system^15^. Temporary confinement will be a family or community response to the process of diagnosis, thus having an impact on how the case will be managed. The ETC will be the referral site after temporary containment of those who test positive for EVD. The advantage of such temporary containment is that it will not take longer than the time for testing and diagnosis. The devices used for testing and diagnosis should be as minimal as possible. The overflow of vehicles to a specific location draws the attention of community members, who may even anticipate the outcome based on rumors related to the activity around the TCA. Temporary containment will help reduce the stigma around suspected cases, as potential EVD-positive patients will not be in the public eye.

Throughout this research, the people we met emphasized the importance of accountability by community members. Temporary confinement will contribute to increasing the responsibility of community members, as people whose involvement is requested are relatives or close friends of the suspected cases. As such, they will not feel committed to a task or activity for others but for themselves. We propose that it is easier for community members to find the motivation to get involved, because of their established relationship with the person who is a suspected EVD case. As such, the immediate benefit of involvement is more evident. Proximity and familiarity with the people involved in the process are essential elements; they are a source of trust. Furthermore, traditional therapists are proximate and familiar, among other reasons for use, and should also be used in the testing and diagnosis of EVD. They are known and considered by the community members as one of their own.

However, the implementation of temporary containment must also be accompanied by more discreet measures. Everything must be done in a medically safe space, with appropriate infection prevention and control measures. All stakeholders, such as health providers and family or community members, must be discreet to effectively prevent the spread of EVD.

## Conclusion

The current healthcare system has been established to care for people with EVD symptoms. First, people are admitted to a TC, where examinations are conducted. If the case turns out to be negative, the person is taken out of the healthcare system; if the case is positive and there is confirmation of the disease, the person is referred to an ETC. A TC is a physical structure, aimed to relieve the burden on ETCs. However, community members fear that TCs are just like ETCs. Misconceptions regarding TCs discourage people from adhering to the treatment plan provided. TCs are seen as a place to go for treatment, rather than testing and diagnosis. Some community members even refuse to allow their relatives to be taken there. People admitted to TCs sometimes flee before being tested or before their test results become known by the community, which creates an issue for healthcare personnel. Health facilities are often deserted during the EVD epidemic. This situation does not favor the use of TCs, which are considered integral to the control and treatment of EVD.

Throughout this research, people who had been suspected of contracting EVD were more willing to stay at home for any examinations rather than going to a TC. The fundamental reason involved discretion and fear of being stigmatized. According to the participants, once a person is admitted to the TC, rumors begin to circulate regarding the diagnosis, and the person starts to be seen by the public as part of the care circuit, even before a positive result is established.

There is, however, an alternative to TCs. It is a matter of finding a space where the potentially ill people are examined. This space should be decided among the family members, who will then become responsible for their symptomatic relatives. Home-based care should be designed by the families and submitted to the health personnel for approval. Necessary arrangements should be made to allow testing under the required conditions. The person’s home will then become a TCA. This will reduce fear and stigma among community members.

Isolation has several advantages, such as discretion and privacy, which prevents stigmatization. The setting up of a TCA also decentralizes the care for people with symptoms. It requires accountability and involvement of the family members, as was the case for the home-based management of malaria.

## Figures and Tables

**Table 1:** Distribution of participants in the in-depth interview (IDI) and focus group discussion (FGD) sessions by provinces

Target	North Kivu				Ituri Province			
	Butembo		Beni		Mbuti		Bunia	
	IDI	FGD	IDI	FGD	IDI	FGD	IDI	FGD
Pillar leads	All		All		All		All	
Pillar members	2/pillar		2/pillar		2/pillar		2/pillar	
Community leaders^1^	≥2/community		≥2/community		≥2/community		≥2/community	
Leader of survivor group	≥2/community		≥2/community		≥2/community		≥2/community	
Community adult males		≥2 groups		≥2 groups		≥2 groups		≥2 groups
Community adult females		≥2 groups		≥2 groups		≥2 groups		≥2 groups
Community male youth		≥2 groups		≥2 groups		≥2 groups		≥2 groups
Community female youth		≥2 groups		≥2 groups		≥2 groups		≥2 groups
Survivors		≥2 groups		≥2 groups		≥2 groups		≥2 groups

1 The community leaders include traditional, religious, political, and social opinion leaders.

## Data Availability

The data that support the findings of this study are not publicly available due to containing information that could compromise the privacy of the research participants. The data are available from the corresponding author upon reasonable request.
